# Boronic acid disk diffusion for the phenotypic detection of polymerase chain reaction-confirmed, carbapenem-resistant, gram-negative bacilli isolates

**DOI:** 10.1186/s12866-016-0754-z

**Published:** 2016-07-01

**Authors:** Rasha Elsherif, Dalia Ismail, Sanaa Elawady, Samyah Jastaniah, Saad Al-Masaudi, Steve Harakeh, Gamal Karrouf

**Affiliations:** Clinical and Chemical Pathology Department, Faculty of Medicine, Cairo University, Giza, Egypt; Biology Department, Faculty of Science, King Abdulaziz University, Jeddah, Saudi Arabia; Special Infectious Agents Unit-Biosafety Level 3, King Fahd Medical Research Center, King Abdulaziz University, Jeddah, 21589 Saudi Arabia; Medical Physics Department, Faculty of Science, King Abdulaziz University, Jeddah, 21589 Saudi Arabia; Surgery, Anesthesiology and Radiology Department, Faculty of Veterinary Medicine, Mansoura University, Mansoura, 35516 Dakahlia Egypt

**Keywords:** Boronic acid, Carbapenem, Disk diffusion *Enterobacteriaceae*, Phenotypic screening

## Abstract

**Background:**

The Middle East is regarded as a secondary reservoir for OXA-48 and New Delhi metallo-β-lactamase (NDM) carbapenemases. One of the main challenges in clinical microbiology diagnostics is the detection of carbapenemases. For this reason simple screening methods have been sought to detect carbapenemase producers to determine appropriate therapeutic measures and implement infection control interventions. The present study aimed to evaluate the efficacy of the modified Hodge test (MHT) and a boronic acid-based combined disk test using carbapenems as substrates for the phenotypic determination of OXA-48 and NDM type carbapenemases in 45 epidemiologically unrelated carbapenem-resistant clinical isolates of *Klebsiella pneumoniae* (13 isolates), *Acinetobacter baumanii* (20 isolates), and *Pseudomonas aeruginosa* (12 isolates).

**Results:**

Boronic acid disk test using meropenem as substrate and 600 µg of 3- aminophenylboronic acid (APB) was the most sensitive method (83.33 %) for detection of OXA-48, while the most specific method was MHT (100 %). As regards NDM carbapenemase, boronic acid disk tests using imipenem and 600 µg of APB per disk, and meropenem with 300 or 600 µg of APB were the most  sensitive methods (87.50 %), while the most specific method was the MHT (100 %).

**Conclusions:**

The results of the present study indicate that phenotypic screening with the MHT and the boronic acid disk test may be used to detect OXA-48 and NDM carbapenemases in Gram-negative bacilli clinical isolates, and that these tests can be easily applied in tertiary care settings with minimal infrastructure.

## Background

The emergence of carbapenemase-producing, Gram-negative bacilli (GNB) has become an emerging public health problem worldwide [1]. The emergence of GNB may lead to variable levels of carbapenem resistance, as well as to resistance to all β-lactam drugs, thereby leading to fewer options for treating such infections, which have mortality rates as high as 50 % [2, 3]. Moreover, carbapenemases-encoding genes are harbored in genetically mobile elements, which allows their rapid spread between GNB [4]. It has been postulated that the spread of extended-spectrum β-lactamase (ESBL) producers was an important factor that led to the increased use of carbapenems, which has enhanced the selection of carbapenemase producers [5, 6].

The currently widespread carbapenemases are the rapid class A carbapenemases of the *Klebsiella pneumoniae* carbapenemase (KPC) type, the class B carbapenemases of the New Delhi metallo-β-lactamase (NDM)-1 type, the imipenem (IMP), and the Verona integron-encoded metallo-β-lactamase types, and the class D carbapenemases of the OXA-48 type [2]. The Middle East is regarded as a secondary reservoir for OXA-48 and NDM carbapenemases [6, 7]. Indeed, the introduction of some OXA-48 and NDM expressing *Enterobacteriaceae* in some European countries originated from hospital patients that were previously hospitalized in Egypt [8–11].

In view of the alarming increase in the appearance of carbapenemase-producing bacteria in clinical isolates in Egypt, a standard testing method should be used for their detection to enable a suitable course of therapy to be followed as part of an infection control program [12]. Molecular methods offer high sensitivity and specificity and a rapid turnaround time. However, they cannot be routinely used in countries with limited resources and a high level of carbapenem resistance.

Phenotypic tests based on the inhibitory activity of boronic acid compounds are easy to perform and interpret. Boronic acid compounds are serine-type-β-lactamase inhibitors that are not based on the β-lactam structure. They are known as class C enzyme inhibitors. Boronic acid tests using cefoxitin, cefotaxime, and ceftazidime disks were used successfully detect AmpC enzymes [13]. Subsequently, boronic disk tests using carbapenems have been proposed to be accurate phenotypic tests for KPC [13, 14].

Because the value of the modified Hodge test (MHT) and a boronic acid-based combined disk test for the determination of widespread carbapenemase producers (NDM-1and OXA-48) has been poorly documented our study to used a collection of OXA-48 and NDM producers as well as carbapenemase non-producers. The aim of the present study was to examine the accuracy of the MHT and a boronic acid-based combined disk test using carbapenems as substrates in the phenotypic determination of OXA-48 and NDM carbapenemases, and to study the behavior of ESBL producers during such tests. Additionally, we aimed to evaluate the performance of a boronic acid combined disk test using ceftazidime (30 μg) as well as two concentrations of 3-aminophenylboronic acid (APB; 300 and 600 μg), as substrates for the phenotypic detection of AmpC enzyme production.

## Methods

### Ethics statement

The present study was approved by the Faculty of Medicine Cairo University Hospital, Egypt. Written informed consent was not necessary for this retrospective study, as it was part of our standard microbiological routine. Patient data were anonymous for the purposes of this analysis, and all confidential patient information was protected in accordance with Egyptian law.

### Hospital setting

The study was conducted at Cairo University Hospital, which serves patients in Cairo (Egypt) and provides medical and surgical care in all medical specialties. The study took place from March to August 2013. The study conforms to the relevant regulatory standards and is in accordance with the recommendations of the Clinical and Laboratory Standards Institute (CLSI) guidelines.

A total of 45 epidemiologically unrelated carbapenem resistant *K. pneumoniae*, *Acinetobacter baumanii*, and *Pseudomonas aeruginosa* isolates obtained from a surgical intensive care unit were included in the study. The isolates included 32 carbapenemase producers {OXA-48 (*n* = 24), NDM (*n* = 8)}. The strains had been characterized previously by the polymerase chain reaction (PCR). None of the isolates were KPC producers. To verify the specificity of the methods for detecting OXA-48 and NDM type carbapenemases, 13 carbapenem-resistant GNBs (six *A. baumanii,* four *P. aeruginosa,* and three *K. pneumoniae* isolates) were chosen for testing. All 13 isolates were negative for the *bla*_OXA-48_, *bla*_NDM_, and *bla*_KPC_ genes.

### Antimicrobial susceptibility of isolates, and screening of phenotypes

A detailed antimicrobial (IMP, meropenem, cefoxitin, ceftazidime, cefpodoxime, ceftriaxone, amikacin, gentamicin, trimethoprim–sulfamethoxazole, polymyxin B, colistin, tigecycline, and fluoroquinolones (Becton Dickinson, Sparks, MD, USA) susceptibility analysis was conducted using the disc diffusion method according to CLSI guidelines.

### Phenotypes of detected ESBLs

ESBL screening was conducted via the disk diffusion test using ceftazidime (30 μg) and cefpodoxime (10 μg) in accordance with CLSI guidelines. Confirmation of the ESBL phenotype was determined using the double synergy test according to CLSI guidelines [15].

### Phenotypic detection of ambler class C β-lactamase

Resistance to cefoxitin (30 μg) was used for the presumptive identification of AmpC β-lactamase [16].

### Boronic acid disk tests

The isolates were suspended and diluted in normal saline to 10^8^ colony-forming units (CFU)/ml by comparison with a McFarland 0.5 turbidity standard, and spread onto Mueller–Hinton agar plates (Mast Diagnostics, Merseyside, UK) as recommended by the CLSI. The following disks (Mast Diagnostics) were tested: IMP (10 μg), IMP (10 μg) with APB (300 μg), meropenem (10 μg), meropenem (10 μg) with APB (300 μg), ceftazidime (30 μg), ceftazidime (30 μg) with APB (300 μg), IMP (10 μg), IMP (10 μg) with APB (600 μg), meropenem (10 μg), meropenem (10 μg) with APB (600 μg), ceftazidime (30 μg), and ceftazidime (30 μg) with APB (600 μg). APB (Sigma-Aldrich, St. Louis, MO, USA) was dissolved in water at 50 mg/ml, and 6 and 12 μl (for the 300 and 600 μg concentrations, respectively) was applied per disk. A 5-mm difference in zone diameter was used as a cutoff to identify resistant isolates [14].

### The MHT

The MHT was performed to confirm the production of carbapenem-hydrolyzing β-lactamases in accordance with CLSI guidelines. MHT Positive Klebsiella pneumoniae ATCC1705 and MHT Negative Klebsiella pneumoniae ATCC1706 were used as quality control for the test.

### Detection of resistance genes

PCR amplification was used to detect carbapenemase genes (*bla*_KPC_, *bla*_NDM_, and *bla*_OXA-48_) using previously described primers and methodology [17–19].

### Mass spectrometry

A pure, single colony was directly deposited on a matrix-assisted laser desorption/ionization time-of-flight (MALDI-TOF), reusable, polished steel target plate (Bruker Daltonik GmbH, Bremen, Germany), and one such deposit was made for each isolate. The preparation was overlaid with 1 μL of matrix solution (a saturated solution of α-cyano-4-hydroxycinnamic acid powder dissolved in a standard solvent: 50 % acetonitrile, 2.5 % trifluoroacetic acid, and 47.5 % deionized water), and the matrix-sample was crystallized in the analyte molecules at room temperature and analyzed within 24 h. Measurements were performed with a MALDI-TOF mass spectrometer (Bruker Daltonik GmbH) using Bruker MALDI Biotyper RTC (Real Time Classification) software version 3.1. The 15 bacterial species exhibiting peptidic patterns that were most similar to that of the isolate were ranked by their identification score.

### Criteria for the identification of isolate

We used the score values proposed by the manufacturer. Meaning of score values (standard sample) and color of range description symbols: 2.300–3.000, highly probable species identification (+++) green); 2.000–2.299, secure genus identification (probable species identification (++) green); 1.700–1.999, probable genus identification ((+) yellow); 1.699, unreliable identification ((–) red).

### Statistical methods

Data were coded and entered using the statistical package SPSS Statistics for Windows, Version 21.0 (IBM Corp., Armonk, NY, USA). Data was summarized using frequency (count) and relative frequency (percentage) for categorical data. To compare categorical data, a chi-square (χ2) test was performed. An exact test was used when the expected frequency was less than 5. The kappa measure of agreement was used to determine the agreement between measures. Sensitivity, specificity, positive likelihood ratio (LR+), and negative likelihood ratio (LR−), as well as their 95 % confidence intervals (CIs), were calculated. *P*-values less than 0.05 were considered to be statistically significant.

## Results

### Clinical isolates

**Fig. 1:**
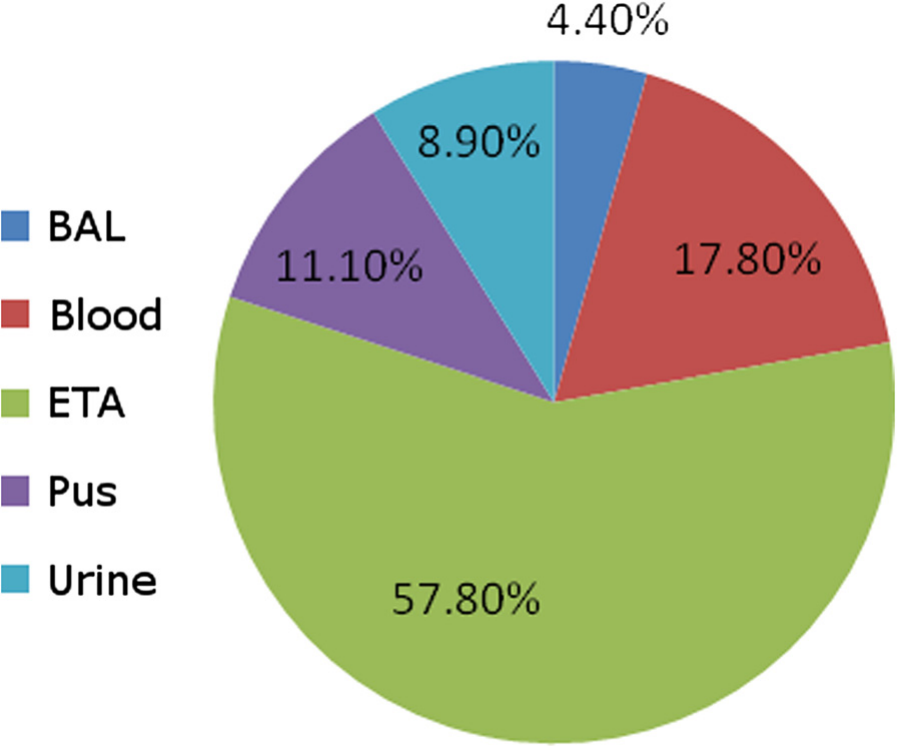
The distribution of specimens in the study; BAL: bronchoalveolar lavage; ETA: endotracheal aspirate

**Fig. 2:**
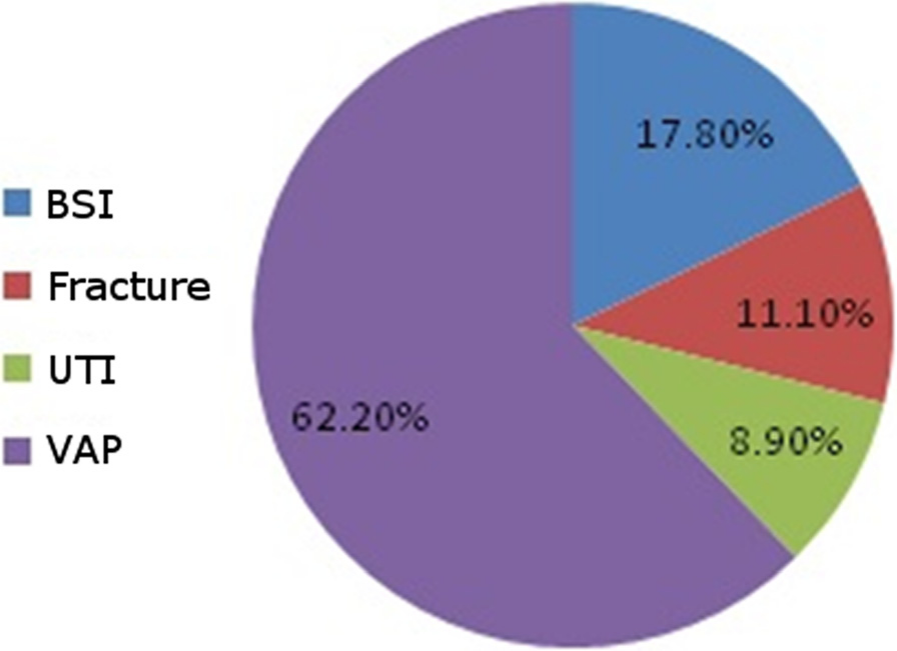
The clinical diagnosis associated with the isolates; BSI: blood stream infection; UTI: urinary tract infection; VAP: ventilator associated pneumonia

**Fig. 3:**
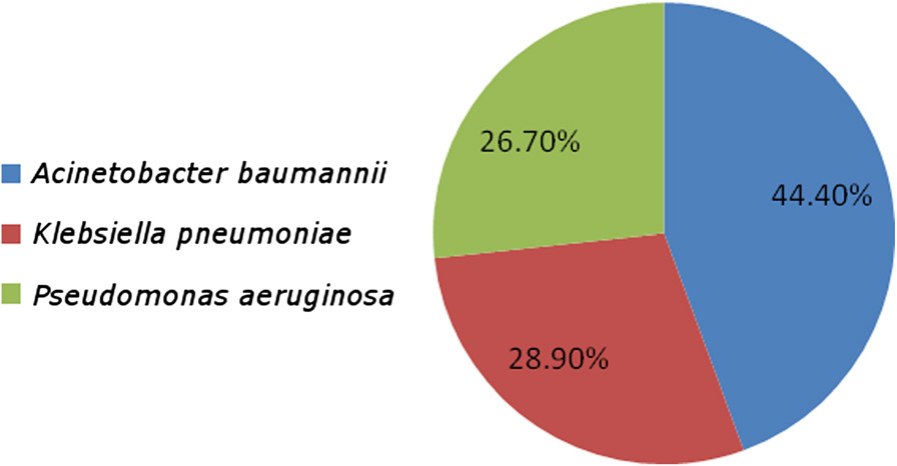
The distribution of isolates in the study; the commonest were *Acinetobacter baumannii* isolates: 20 (44.40 %), followed by *Klebsiella pneumoniae*: 13 (28.90 %), and *Pseudomonas aeruginosa* 12 (26.70 %)

**Fig. 4:**
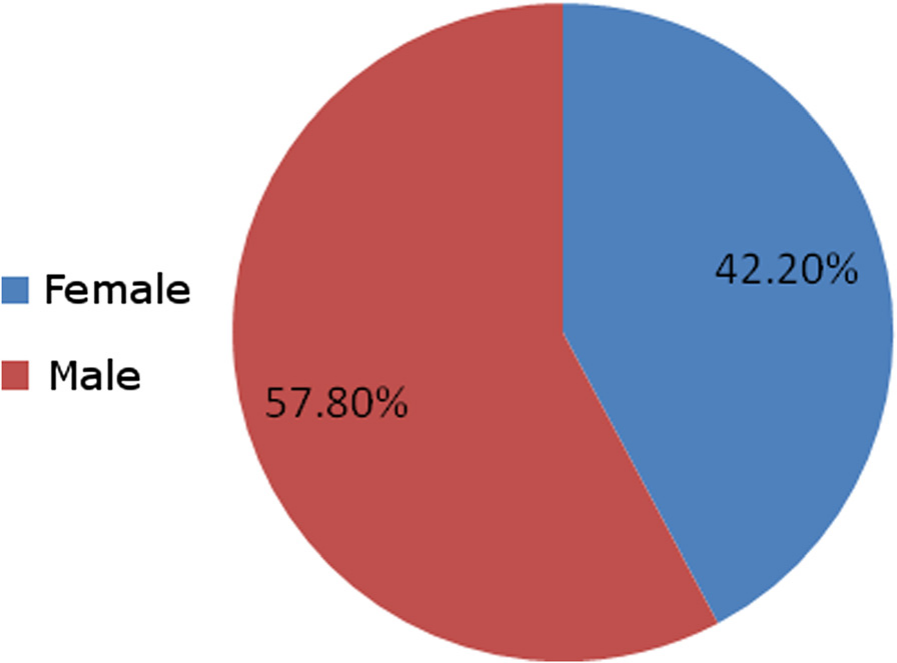
The gender distribution in this study male 57.80 % and female 42.20 %

Among the 45 carbapenem-resistant GNB isolates, 28 came from respiratory tract specimens (62.2 %), eight from blood (17.8 %), five from wounds (11.1 %), and four from urine (8.9 %) (Fig. 1). The most common routes of infection of these organisms were ventilator-associated pneumonia (62.2 %), followed by bloodstream infections (17.8 %), fractures (11.1 %), and urinary tract infections (8.9 %) (Fig. 2). The most common isolates were: *A. baumannii* (20, 44.40 %), followed by *K. pneumoniae* (13, 28.90 %), and *P. aeruginosa* (12, 26.70 %) (Fig. 3). The male:female ratio in this study was 1:1.36, with males and females constituting 57.80 and 42.20 %, respectively, of the patients (Fig. 4). The results of the antimicrobial sensitivity tests were as follows: 20 % of the isolates were sensitive to amikacin; 57.8 % were sensitive to polymyxin B; 37.8 % were sensitive to tigecycline; 22 % were sensitive to ceftazidime; 2.2 % were sensitive to trimethoprim–sulfamethoxazole; 2.2 % were resistant to all tested antibiotics; 86.66 % were resistant to cefoxitin; and 100 % were resistant to IMP, meropenem, ceftazidime, cefpodoxime, ceftriaxone, and gentamicin. All isolates met the ESBL screening and confirmatory test criteria, as they demonstrated reduced susceptibility to ceftazidime (30 μg) and cefpodoxime (10 μg) and yielded positive results in the double-disk synergy test.

### Phenotypic detection of OXA-48 producers

The results of the MHT are summarized in Table 1. The MHT was positive for 19 OXA-48-producing clinical isolates, thus confirming the presence of significant carbapenem-hydrolyzing activity. Five of the OXA-48 isolates yielded negative results with the MHT (79.17 % sensitivity). All non-carbapenemase producers yielded negative results with the MHT (100 % specificity) (Table 1).

**Table 1:** Results of MHT and boronic acid disk test for the phenotypic detection of OXA-48 producers

OXA-48 producers	MHT		BA disk test	
	Positive	Negative	Positive	Negative
*Acinteobacter baumannii* (11)	9	2	10	1
*Pseudomonas aeruginosa* (6)	4	2	3	3
*Klebisella pneumoniae* (7)	6	1	7	0

### Boronic disk test

The results using the boronic disk test are summarized in Table 2. Twenty OXA-48-producing isolates tested positive using the boronic disk test (using meropenem and 600 μg of APB). Four of the 24 OXA-48 isolates yielded negative results with the boronic disk test (87.50 % sensitivity). Three of the carbapenemase non-producers yielded false-positive results with the boronic disk test (using IMP and 300 μg of APB). The highest sensitivity (83.33 %) was noted when using meropenem with 600 μg of APB. The highest specificity (76.92 %) was observed when using IMP with 300 μg of APB (Table 2).

**Table 2:** Summary of sensitivities, specificities, positive and negative predictive values of the boronic acid disk test using different antibiotic substrates with different concentrations of APB, for the phenotypic detection of OXA-48 producers

Antibiotic used in boronic acid disk test	No. of isolates confirmed by PCR as		Test performance			
	Possessing OXA-48 (*n* = 24)	Not possessing OXA-48 (*n* = 13)	Sensitivity	Specific	Positive predictive value	Negative predictive value
IPM 300	17	3	70.8 %	76.92 %	85 %	58.82 %
MEM 300	18	8	75 %	38.46 %	69.23 %	45.45 %
IPM 600	19	8	79.17 %	38.46 %	70.37 %	50 %
MEM 600	20	9	83.33 %	30.77 %	68.97 %	50 %

### Phenotypic detection of NDM producers

Seven NDM-producing clinical isolates tested positive using the MHT, thus confirming the presence of significant carbapenem-hydrolyzing activity. One of the eight NDM isolates yielded negative results with the MHT (87.50 % sensitivity). All of the carbapenemase non-producers yielded negative results with the MHT (100 % specificity) (Table 3).

**Table 3:** Results of MHT and boronic acid disk test for the phenotypic detection of of NDM producers

OXA-48 producers	MHT		BA disk test	
	Positive	Negative	Positive	Negative
*Acinetobacter baumannii* (3)	2	1	3	0
*Pseudomonas aeruginosa* (3)	2	1	3	0
*Klebisella pneumoniae* (2)	2	0	1	1

### Boronic acid disk test

Seven NDM-producing isolates tested positive using the boronic acid disk test. One of the eight NDM-producing isolates yielded negative results with the boronic acid disk test (87.5 % sensitivity). Three of the non-carbapenemase producers yielded false-positives with the boronic acid disk test (IMP with 300 μg of APB) (76.92 % specificity). The highest sensitivity (87.50 %) was noted using meropenem with 300 μg of APB, meropenem with 600 μg of APB, and IMP with 600 μg of APB (Table 4).

**Table 4:** Summary of sensitivities, specificities, positive and negative predictive values of the boronic acid disk test using different antibiotic substrates with different concentrations of APB, in the phenotypic detection of NDM producers

Antibiotic used in boronic acid disk test	No. of isolates confirmed by PCR as		Test performance			
	Possessing NDM (*n* = 8)	Not possessing NDM (*n* = 13)	Sensitivity	Specific	Positive predictive value	Negative predictive value
IPM 300	6	3	75 %	76.92 %	66.67 %	83.33 %
MEM 300	7	8	87.5 %	38.46 %	46.67 %	83.33 %
IPM 600	7	8	87.5 %	38.46 %	46.67 %	83.33 %
MEM 600	7	9	87.5 %	30.77 %	43.75 %	80 %

### Phenotypic detection of AmpC enzyme producers using the boronic disk test

Of the 45 carbapenem resistant GNB, 39 (86.66 %) were phenotypically identified as AmpC producers by ceftazidime screening. Boronic acid disk tests using ceftazidime as a substrate were positive for 29 and 33 of the Fox-resistant isolates when using 300 and 600 μg of APB, respectively (sensitivities of 74.36 and 84.36 %, respectively). None of the Fox-sensitive isolates yielded positive results with the test (100 % specificity) (Table 5).

**Table 5:** Results of boronic acid disk tests using ceftazoidime (CAZ) as substrate with either 300 or 600 μg of APB in the phenotypic detection of Amp C

	For screening				Test performance			
	FOX resistant (*n* = 31)		FOX sensitive (*n* = 6)		Sensitivity	Specificity	PPV	NPV
	+ve by boronic acid	-ve by boronic acid	+ve by boronic acid	-ve by boronic acid				
CAZ 300	29	10	0	6	74.36 %	100 %	100 %	37.5 %
CAZ 600	33	6	0	9	84.67 %	100 %	100 %	50 %

## Discussion

OXA-48 and NDM enzymes have become increasingly prevalent among GNB isolates in North Africa and the Middle East [7, 8]. Given the limited therapeutic options available, the accurate detection of these enzymes is the crucial first step in controlling their spread and ensuring optimal clinical outcomes. Real-time or multiplex PCR analyses may accurately identify such isolates, but these methods are not suitable for daily testing in clinical laboratories because of their high cost and inconvenience [20]. Thus, simple, cost-effective techniques have been sought. In the current study, the MHT and a boronic acid disk test were tested against a collection of GNB clinical isolates, some of which tested positive and negative for OXA-48 and NDM using PCR as the standard.

The MHT and boronic acid disk tests have been reported to be accurate assays for the phenotypic detection of KPC carbapenemases [13, 20–22]. However, for carbapenemases other than KPCs, the data regarding the utility of these tests are unsatisfactory [23]. Regarding the MHT, studies have come to contrasting conclusions, with some showing it to be inadequate for detecting metallo-β-lactamases [5] and OXA-48 [23], while others showed that the MHT produces false-positive results for carbapenemases [3, 5]. The CLSI published a recommendation that stated that *Enterobacteriaceae* with elevated carbapenem minimum inhibitory concentrations or reduced disk diffusion inhibition zones should be tested for carbapenemase production using the MHT; however, this recommendation does not include *P. aeruginosa* [24].

Boronic acid compounds have been shown to be excellent AmpC inhibitors [13] Subsequently, they have been shown to be excellent KPC inhibitors [24]. In the present study, we evaluated the use of boronic acid disk tests for the phenotypic detection of OXA-48- and NDM-producing GNB isolates in the clinical laboratory. The inhibitory activity of APB (300 and 600 μg) with IMP and meropenem as antibiotic substrates was tested against a collection of clinical isolates.

The clinical isolates included 32 carbapenemase producers (24 OXA-48 producers and eight NDM producers) and 13 carbapenemase non-producers. For the 24 OXA-48 producers, the sensitivity of the MHT was 79.17 %; the average reported sensitivity of the MHT is >90 % [22]. The low sensitivity in our study could be attributed to the fact that two *P. aeruginosa* isolates and two *A. baumannii* isolates were not detected by the MHT, or to the failure of the MHT to detect carbapenemase activity in two *P. aeruginosa* isolates and two *A. baumannii* isolates, as well as in one *K. pneumoniae* isolate. This is in accordance with a report by Pasteran et al. [25], who stated that the MHT is not suitable for detecting carbapenemase production in *P. aeruginosa*, as the reported sensitivity and specificity were 78 and 57 %, respectively. Furthermore, a study in India concluded that the MHT is not preferred for carbapenemase detection in non-fermenting GNB. Moreover, the CLSI recommendation for the MHT applies to all *Enterobacteriaceae*, except *P. aeruginosa* [24]. For the eight NDM producers, the MHT was positive, except for one *A. baumannii* isolate (87.50 % sensitivity). All carbapenemase non-producing species yielded negative results with the MHT (100 % specificity).

In the current study, boronic acid disk tests using IMP and meropenem as antibiotic substrates, with 300 and 600 μg of APB, demonstrated variable results regarding the differentiation of carbapenemase producers. For the 24 OXA-48 isolates, the boronic acid disc test was positive for 20 isolates using meropenem as a substrate with 600 μg of PBA. Five isolates (two *A. baumannii* isolates, two *P. aeruginosa* isolates, and one *K. pneumoniae* isolates) yielded false-negative results. All of the other *K. pneumoniae*, *P. aeruginosa*, and *A. baumannii* isolates were accurately identified. The highest sensitivity (83.33 %) was obtained using a meropenem disk combined with 600 μg of APB. The boronic disk test was positive for all NDM-producing isolates, except one *A. baumannii* isolate (87.50 % sensitivity), and all of the other *K. pneumoniae*, *P. aeruginosa*, and *A. baumannii* isolates were accurately identified. False-positive results were observed for carbapenemase non-producers using the boronic acid disk test. The highest specificity (76.92 %) was achieved using an IMP disk with 300 μg of APB. False-positive results among these isolates could be attributed to the concomitant production of the AmpC β-lactamase (12 of the 13 carbapenemase non-producers were AmpC producers). This is in accordance with the results of Giske et al. [26] who attributed the false-positive results of the boronic acid disk test in their isolates to AmpC production, coupled with porin loss.

ESBL production was detected in 100 % of our isolates. This is in accordance with the results obtained in Egypt by Zafer et al. [27], who found that 100 % of multidrug-resistant *Enterobacteriaceae* isolates were ESBL producers. Overall, the boronic acid disk test using meropenem as a substrate with 600 μg of APB was the most sensitive method (83.33 %) for the detection of OXA-48, while the most specific method was the MHT (100 %). Concerning NDM carbapenemase, the boronic acid disk test using IMP with 600 μg of APB, and meropenem with 300 or 600 μg of APB were the most sensitive methods (87.50 % each), while the most specific method was the MHT (100 %). Nevertheless, the MHT and boronic acid disk test are convenient assays for the initial screening of potential OXA-48 and NDM producers among carbapenem-resistant GNB [13, 14]. However, the validity of such tests in non-fermenters needs to be confirmed.

The boronic acid test using disks of ceftazidime with 600 μg of APB was also sensitive and specific for the detection of AmpC producers (sensitivity, 84.36 %; specificity, 100 %). This result is in accordance with a study by Coudron et al. [16], who stated that the boronic acid disk test is a simple and efficient method for detecting plasmid-mediated AmpC production. Furthermore, the boronic acid disk test also enhanced the detection of isolates that harbored both ESBLs and AmpC β-lactamases, which was also the case in our study. However, this test failed to detect AmpC production in six resistant isolates (five AmpC-producing *P. aeruginosa* isolates and one AmpC-producing *K. pneumoniae* isolate). For the other *K. pneumoniae*, *A. baumannii*, and *P. aeruginosa* isolates, AmpC production was accurately detected using ceftazidime and 600 μg of APB (97.05 % sensitivity). This finding is in accordance with a report by Upadhyay et al. [28], who evaluated several inhibitor-based methods for the detection of AmpC production in *P. aeruginosa*, none of which could detect all of the AmpC variants of clinical importance. They undertook a study using a variety of inducers and inhibitors, including the boronic acid inhibition test, and the sensitivity was 43 %.

In recent years, Egypt has been among the countries with the highest reported rates of antimicrobial resistance [27]. Plasmid-mediated, AmpC-producing isolates are frequently detected (86.66 % of the isolates in the current study), and in several cases they contribute to reduced susceptibility to carbapenems, which was observed among AmpC producers that did not harbor carbapenemase-encoding genes, as determined by PCR, in the present study; this finding is also supported by a study by Noyal et al. [29]. Our study is limited by the relatively small number of isolates assessed. However, replicating the study using a large number of diverse samples of OXA-48 and NDM producers and non-producers will help to establish the reliability of these tests.

## Conclusions

The results of this study clearly demonstrate that phenotypic screening with the MHT and boronic acid disk test may have an important role in the detection of OXA-48 and NDM carbapenemases in GNB clinical isolates, and these tests, which are cost-effective, can be easily applied in any tertiary care settings with minimal infrastructure. Routine testing of all carbapenem-resistant clinical isolates for possible carbapenemase activity may increase the availability of data for such isolates, as only a few studies have examined this phenomenon. Confirmation of the resistance mechanism is not required from a public health perspective.

## Abbreviations

AK, Amikacin; APB, aminophenylboronic acid; AST, Antibiotic sensitivity test; BA, boronic acid; CFU, Colony-forming units; CIP, Ciprofloxacin; CLSI, Clinical and Laboratory Standards Institute; ESBL, Extended Specteram Beta-Lactameses; ETA, Endotracheal aspirate; GNB, Gram-negative bacilli; ICU, Intensive Care Unit; IMP, Imipenem; KPC, *Klebsiella pneumoniae* carbapenemase; MALDI-TOF, Matrix associated laser desorption-ioinization time of light; MHT, Modified Hodge test; NDM, New Delhi metallo-β-lactamase; NPV, Negative predictive value; PB, polymyxin B; PCR, polymerase chain reaction; PPV, Positive predictive value; TGC, Tigycyclin; Ts, trimethoprim–sulfa methoxazole.
